# Effect of bevacizumab plus XELOX (CapeOX) chemotherapy on liver natural killer cell activity in colorectal cancer with resectable liver metastasis

**DOI:** 10.1002/ags3.12195

**Published:** 2018-07-18

**Authors:** Fumihiro Hirata, Kohei Ishiyama, Yuka Tanaka, Tsuyoshi Kobayashi, Masakazu Hashimoto, Yoshihiro Saeki, Nobuki Ishida, Kazuhiro Taguchi, Junko Tanaka, Koji Arihiro, Hideki Ohdan, Hiroshima Surgical Study Group of Clinical Oncology (HiSCO)

**Affiliations:** ^1^ Department of Gastroenterological and Transplant Surgery Graduate School of Biomedical & Health Sciences Hiroshima University Hiroshima Japan; ^2^ Department of Surgery National Hospital Organization Kure Medical Center and Chugoku Cancer Center Hiroshima Japan; ^3^ Department of Epidemiology Infectious Disease Control and Prevention Graduate School of Biomedical & Health Sciences Hiroshima University Hiroshima Japan; ^4^ Department of Anatomical Pathology Hiroshima University Hospital Hiroshima Japan

**Keywords:** chemotherapy, colorectal cancer with liver metastasis, liver immunity, natural killer cell

## Abstract

**Aim:**

We investigated the chemotherapy effect of resectable colorectal cancer with liver metastasis (CRLM) on the function of intrahepatic immune cells.

**Methods:**

We classified patients into adjuvant chemotherapy (bevacizumab+CapeOX) after hepatectomy group (group A) and neoadjuvant chemotherapy followed by hepatectomy group (group B), and collected peripheral blood mononuclear cells (PBMC) and liver mononuclear cells (LMNC) to ascertain phenotypic and functional differences.

**Results:**

There were no significant differences in lymphocyte fractions of either PBMC or LMNC between groups, except for the significantly lower percentage of natural killer (NK) cells in LMNC in group B than in group A. Significantly higher percentage of natural‐killer group 2, member D (NKG2D)‐ positive NK cells in PBMC and percentage of tumor necrosis factor‐related apoptosis‐inducing ligand (TRAIL)‐, NKp30‐, and signal regulatory protein β (SIRPβ)‐positive NK cells in LMNC were found in group B. Furthermore, significantly higher expressions of NKG2D and SIRPβ in peripheral blood NK cells and of NKp46 and CD122 in liver NK cells were found in group B. When LMNC were incubated with interleukin (IL)‐2 in vitro, no difference was observed in the expression of these molecules in NK cells between groups. Consistently, there was no difference in the cytotoxic activity of those LMNC against a colon adenocarcinoma cell line between groups.

**Conclusion:**

Colorectal cancer with liver metastasis patients treated with neoadjuvant chemotherapy showed enhanced expression of activation markers on peripheral blood and liver NK cells in comparison with patients who did not receive therapy; however, the difference in those function remains unclear. These results suggest that neoadjuvant chemotherapy does not have a negative impact on intrahepatic immune cells in resectable CRLM patients.

## 1 INTRODUCTION

The primary clinical complication of colorectal cancer (CRC) is the invasion of tumor cells into distant organs and outgrowth of metastases. CRC with liver metastasis (CRLM) is a major prognostic factor for CRC patients.^1^, ^2^ Disease‐specific mortality of progressive CRC has decreased significantly in past decades with novel chemotherapeutic agents, such as oxaliplatin and irinotecan, and molecular targeted drugs, such as bevacizumab, a recombinant humanized monoclonal antibody that targets vascular endothelial growth factor (VEGF).^3^ Adding bevacizumab to chemotherapy significantly reduces the residual viable tumor cell volume in resected tumors and increases the proportion of patients eligible for liver metastasis resection, when compared with chemotherapy alone.^4^, ^5^

Despite recent advancements in chemotherapy strategies for the treatment of advanced CRC patients, surgical resection of CRLM has been established as the treatment of choice and is the most effective and potentially curative therapy.^6^, ^7^, ^8^, ^9^, ^10^ Furthermore, surgical resection for CRLM combined with systemic adjuvant chemotherapy has a potential benefit to be curative for CRLM patients.^11^, ^12^ Meanwhile, the advantage of neoadjuvant chemotherapy in initially resectable CRLM patients is the treatment of undetected distant micrometastasis, thereby reducing recurrence risk after surgery.^13^ Although various studies have reported on the treatment strategy in resectable CRLM patients, the optimal treatment sequence remains unclear.

Considering the impact of both surgical resection and chemotherapy on host immunity is imperative in the treatment of resectable CRLM. Immune systems surrounding cancer cells are known to play crucial roles in regulating cancer cell proliferation, invasion, and metastasis through immunosurveillance.^14^, ^15^ Our group and other researchers have shown that the decreased activity of immune cells in the liver after hepatectomy leads to tumor growth in mice.^16^, ^17^ Although the influence of surgery on immune surveillance against cancer cells has been occasionally investigated, the influence of chemotherapy on that remains to be elucidated. Studies have reported that the presence of tumor‐infiltrating immune cells, such as natural killer (NK) cells and T cells, in CRLM patients improved the overall survival (OS).^6^, ^18^ NK cells are part of the innate immune system and may provide a first line of defense against neoplastic cells by exerting an effector function without the necessity for priming.^19^ In addition, NK cells are abundant in human liver, and liver NK cells have remarkably higher cytotoxic activity against neoplastic cells than peripheral blood NK cells.^20^, ^21^ Such unique anatomical distribution and functional property of NK cells in the liver prompt us to investigate the influence of chemotherapy, particularly neoadjuvant chemotherapy, on the immunity of NK cells in the liver of CRLM patients.

In a clinical setting, we are conducting a phase II/III randomized clinical trial in Hiroshima Surgical Study Group of Clinical Oncology (HiSCO) to determine whether neoadjuvant chemotherapy followed by hepatectomy is superior to adjuvant chemotherapy after hepatectomy in resectable CRLM patients regarding progression‐free survival (PFS), OS, and time to treatment failure. As this clinical trial provided a good opportunity to obtain samples from resectable CRLM patients, who were either previously exposed or not exposed to chemotherapy, we additionally but separately designed an independent study to investigate the effect of neoadjuvant chemotherapy in resectable CRLM patients on liver NK cell activity as subanalysis of a clinical trial.

## 2 MATERIALS AND METHODS

### 2.1 Study design and procedures

We have been conducting a phase II/III randomized clinical trial in HiSCO (Hiroshima, Japan) registered with the National Review Board (HiSCO‐01, University Hospital Medical Information [UMIN] 00000378) to elucidate whether neoadjuvant chemotherapy, bevacizumab combined with XELOX (CapeOX: capecitabine plus oxaliplatin), followed by hepatectomy is superior to adjuvant chemotherapy after hepatectomy in resectable CRLM patients. From June 2010, a planned cohort of 260 macroscopically resectable CRLM patients who fulfilled the inclusion criteria (Table 1) were randomly assigned to either adjuvant chemotherapy (eight courses of bevacizumab 7.5 mg/kg with capecitabine 2000 mg/m^2^ plus oxaliplatin 130 mg/m^2^) after hepatectomy group (group A) or neoadjuvant chemotherapy followed by hepatectomy group (group B) based on the discretion of the reference physician in the HiSCO group. In group A, adjuvant chemotherapy was given within 8 weeks after hepatectomy. CapeOX therapy was started, of which eight courses (each lasting 3 weeks) were given. Bevacizumab was given in the second and subsequent courses. In group B, neoadjuvant chemotherapy comprised eight courses of bevacizumab plus CapeOX therapy and each course lasted for 3 weeks. Bevacizumab was withdrawn in the final course. Surgical resection was carried out within 2 and 8 weeks after the completion of neoadjuvant chemotherapy.

**Table 1:** Eligibility criteria for patients with colorectal cancer with liver metastasis

Eligibility criteria	
(1)	Primary lesion histologically diagnosed as CRC
(2)	Presence of CRLM of stage H1 or H2
(3)	Metastatic lesion in the liver required less than 60% of liver resection and allowed resection with a microscopically negative margin
(4)	No distant/peritoneal metastasis other than CRLM
(5)	The primary lesion had already been or could be resected with a microscopically negative margin
(6)	No history of local therapy such as radiofrequency ablation or chemotherapy/radiotherapy for the CRLM
(7)	No history of chemotherapy involving the use of oxaliplatin
(8)	The liver disease could be classified as Child‐Pugh class A
(9)	No evident hemorrhage or obstruction arising from CRC
(10)	Patients were aged between 20 and 80 y during enrollment
(11)	Patient's PS was either 0 or 1

CRC, colorectal cancer; CRLM, colorectal cancer with liver metastasis; PS, performance status.

This study was additionally designed as subanalysis of a randomized clinical trial in HiSCO‐01 to investigate the effect of chemotherapy on the functions of intrahepatic immune cells in resectable CRLM patients. From February 2011, we conducted this additional study in 30 consecutive patients. Number of samples was determined on the basis of our previous study, which indicated the relationship between clinical pathology and activation status of liver and peripheral blood NK cells.^22^, ^23^ We calculated sample size to detect the difference among percentage positive NK cells under the assumption that expected difference 20%, SD 15%, alpha level 5% and 80% power, and it was 10 for each group. Assuming a dropout rate of 33%, target sample size was set as 15 for each group. We obtained clinical data, including patients' characteristics, and data obtained by analysis for comparative assessment. The research was conducted in compliance with the Declaration of Helsinki published by the World Medical Association and the Ethical Guidelines for Clinical Research published by Ministry of Health, Labor, and Welfare, Japan. In addition, this study was approved by the Institutional Review Board (IRB) of all institutions participating in this study. We obtained written informed consent from all patients before enrollment.

### 2.2 Collection of mononuclear cells

We analyzed the contents of immune cells and phenotype using peripheral blood mononuclear cells (PBMC) and liver mononuclear cells (LMNC) obtained from eligible patients in both groups. We collected blood samples at the time of hepatectomy, and PBMC were isolated by gradient centrifugation with Separate‐L (Muto Pure Chemicals Co., Ltd, Tokyo, Japan) from 40 mL heparinized peripheral blood. In addition, LMNC were obtained by ex vivo perfusion through the portal vein of resected livers from CRLM patients as previously described.^20^ Effluents were condensed by centrifugation, and LMNC were isolated by gradient centrifugation with Separate‐L.

### 2.3 Flow cytometric analyses

We carried out flow cytometric (FCM) analyses using a FACSCalibur cytometer (BD Biosciences, San Jose, CA, USA) and FlowJo 7.6.5 software (TreeStar Inc., Ashland, OR, USA). Based on a previous study,^23^ the monoclonal antibodies (mAbs) used for surface staining of lymphocytes to assess the phenotypic properties of NK cells were as follows: fluorescein isothiocyanate (FITC)‐conjugated anti‐CD3 (HIT3a), anti‐CD56 (B159), anti‐CD19 (HIB19); phycoerythrin (PE)‐conjugated anti‐NKp30 (p30‐15), anti‐NKp46 (9E2), anti‐CD122 (Mik‐β3), anti‐CD56 (B159), and anti‐CD11b (ICRF44); and allophycocyanin (APC)‐conjugated anti‐CD3 (HIT3a), anti‐natural‐killer group 2, member D (NKG2D; 1D11), purchased from Becton Dickinson (San Jose, CA, USA); PE‐conjugated anti‐tumor necrosis factor‐related apoptosis‐inducing ligand (TRAIL; RIK‐2; eBioscience, Santa Clara, CA, USA); and PE‐conjugated anti‐signal regulatory protein β (SIRPβ; B4B; BioLegend, San Diego, CA, USA). Mouse immunoglobulin (Ig)G1κ was used as an isotype‐matched control. Dead cells were excluded from the analysis by light scatter analysis and propidium iodide staining.

### 2.4 Cytotoxicity assays

Liver mononuclear cells were suspended in DMEM medium (Gibco, Grand Island, NY, USA) supplemented with 10% heat‐inactivated fetal calf serum (Sanko Chemical Co., Tokyo, Japan), 25 mmol/L HEPES buffer (Gibco), 50 μmol/L mercaptoethanol (Katayama Chemical Co., Osaka, Japan), 50 U/mL penicillin, and 50 μg/mL streptomycin (Gibco). In addition, LMNC were used for phenotypic analyses and cultured with or without human recombinant interleukin (IL)‐2 (100 U/mL; Takeda, Tokyo, Japan) for 3 days at 37°C in 5% CO_2_. Cultured cells were harvested and used for phenotypic analyses and cytotoxicity assays, which were carried out using FACSAriaII (BD Biosciences) and FlowJo 7.6.5 software. Furthermore, we used DLD‐1 cells (Japanese Collection of Research Bioresources Cell Bank, Osaka, Japan), established from a colon adenocarcinoma cell line (Dukes type C), as target cells. FCM assay was carried out to evaluate cell‐mediated cytotoxicity, as described previously^24^ with minor modifications. DLD‐1 cells were labeled with PKH using the PKH26 Fluorescent Cell Linker Kits (Sigma‐Aldrich, St Louis, MO, USA) according to the manufacturer's instructions. PKH‐labeled target cells (1 × 10^5^) and prepared effector cells were added to a total of 200 μL in roundbottom, 96‐well microtiter plates (BD Falcon, San Diego, CA, USA) in duplicate wells. After 4‐hour incubation, cells were harvested and stained with 4′,6‐diamidino‐2‐phenylindole (DAPI; Vector Laboratories, Burlingame, CA, USA). As a control, target cells were incubated in culture medium alone to determine spontaneous cell death. Number of PKH‐labeled target cells that were killed was determined by analyzing PKH and DAPI double‐positive cells using FCM. Cytotoxicity percentage was calculated using the following equation:

$$\begin{array}{cc} & \%\text{cytotoxicity}\\ & =[\left(\%\text{experimental}\;{\text{PKH}}^{+}\;{\text{DAPI}}^{+}\;\text{targets}\right)-(\%\text{spontaneous}\;{\text{PKH}}^{+}\\ & \;{\text{DAPI}}^{+}\;\text{targets})]/\left[100-\left(\%\text{spontaneous}\;{\text{PKH}}^{+}\;{\text{DAPI}}^{+}\;\text{targets}\right)\right]\times 100 \end{array}$$

### 2.5 Real‐time polymerase chain reaction

Total RNA was isolated from resected tumor tissues of CRLM using RNeasy Mini kit (Qiagen, Limburg, the Netherlands) and reverse‐transcribed using ReverTra Ace qPCR RT Kit (Toyobo Life Science, Tokyo, Japan) according to the manufacturer's instructions. We determined the relative copy numbers of NKG2D ligands, such as major histocompatibility complex (MHC) class I polypeptide‐related sequence A (MICA), MHC class I polypeptide‐related sequence B (MICB), and UL16‐binding protein 2 (ULBP2), by real‐time polymerase chain reaction (PCR) using NKG2D ligand‐specific primer pairs and normalized to the expression of glyceraldehyde 3‐phosphate dehydrogenase (GAPDH). We amplified the resulting cDNA with Rotor‐Gene 3000 and Rotor‐Gene SYBR Green PCR Kits (Qiagen) and analyzed the data using ΔCt method for relative quantification. We carried out all RT‐PCR experiments using 2 μL cDNA with the following cycling parameters: 40 cycles at 95°C for 5 seconds and 60°C for 10 seconds. The following primers were used: *MICA*, 5′‐CCTTGGCCATGAACGTCAGG‐3′ (forward) and 5′‐CCTCTGAGGCCTCGCTGCG‐3′ (reverse); *MICB*, 5′‐ACCTTGGCTATGAACGTCACA‐3′ (forward) and 5′‐CCCTCTGAGACCTCGCTGCA‐3′ (reverse); ULBP2, 5′‐CAGAGCAACTGCGTGACATT‐3′ (forward) and 5′‐CATGCCCATCAAGAAGTCCT‐3′ (reverse); and GAPDH (used as an internal control), 5′‐CAACGGATTTGGTCGTATTGG‐3′ (forward) and 5′‐CCATGGGTGGAATCATATTGG‐3′ (reverse).

### 2.6 Image‐confirmed and pathological chemotherapy response rates

We evaluated the image‐confirmed chemotherapy response rate according to the Response Evaluation Criteria In Solid Tumors (RECIST v1.1)^25^ as follows: complete response (CR), partial response (PR), progressive disease (PD), and stable disease (SD). Furthermore, the pathological chemotherapy response rate was defined according to the criteria of the Japanese Society for Cancer of the Colon and Rectum (JSCCR)^10^ as follows: grade 0, grade 1a, grade 1b, grade 2, and grade 3. In this study, the pathologist randomly selected CRLM slides stained with hematoxylin‐eosin, and evaluated chemotherapy effectiveness without knowledge about the clinical history of the patients.

### 2.7 Statistical analysis

Data are presented as mean ± standard deviation (SD). We carried out statistical analyses using JMP 11 for Windows (SAS Institute, Inc., Cary, NC, USA). Statistical significance of the differences observed between groups was evaluated by Mann‐Whitney *U*‐test and ANOVA with Scheffe's *F* test. *P* < 0.05 was considered statistically significant.

## 3 RESULTS

### 3.1 Patient demographics and clinicopathological characteristics

In this prospective open‐label study, we enrolled CRLM patients previously untreated with chemotherapy. In group B, 11 of 15 patients were evaluable for preoperatively given bevacizumab combined with CapeOX at the time of sampling. In group A, 15 patients were used as controls without the influence of chemotherapy. Differences in the number of cases between these both groups were the exclusion of some cases because of complications of chemotherapy, treatment refusal, and another carcinoma occurrence. Table 2 summarizes characteristics of patients in the present study. No significant differences existed in characteristics of patients, including gender, age, synchronous/metachronous tumor, location of primary tumor, clinical stage, tumor differentiation, surgical procedure for hepatectomy, mean resected liver weight, and count of intrahepatic immune cells collected by perfusion between these groups. Synchronous liver resection with primary resection was carried out in one patient in group A (1/10) and in another patient in group B (1/5) with synchronous metastasis. These patients showed no characteristic findings.

**Table 2:** Characteristics of patients in the present study

Patients' characteristics				
		Group A	Group B	*P* value
Eligible patients/enrolled patients		15/15	11/15	
Gender	Male	11	9	1.00
	Female	4	2	
Age (y)	Median (range)	66 (27‐77)	66 (45‐78)	0.78
Relations of CRC and CRLM	Synchronous	10	5	0.43
	Metachronous	5	6	
Location of primary tumor	Colon	9	6	0.82
	Rectum	5	4	
	Colon and rectum	1	1	
Clinical stage	I	0	1	0.55
	II	3	3	
	III	2	2	
	IV	10	5	
Tumor differentiation	Well	2	2	0.66
	Moderately	12	9	
	Poorly	0	0	
	Mucinous	1	0	
Hepatectomy	Partial resection	5	7	0.18
	Segmentectomy	8	2	
	Lobectomy	2	2	
Resection liver weight (g)	Median (range)	183 (26‐845)	200 (10‐638)	0.80
Number of LMNC (×10^6^)	Median (range)	127 (5‐945)	42 (3‐634)	0.34

CRC, colorectal cancer; CRLM, colorectal cancer with liver metastasis; LMNC, liver mononuclear cells.

### 3.2 Neoadjuvant chemotherapy did not affect cell numbers or the proportion of lymphocytes in PBMC and LMNC, except for the proportion of liver NK cells

**Figure 1:**
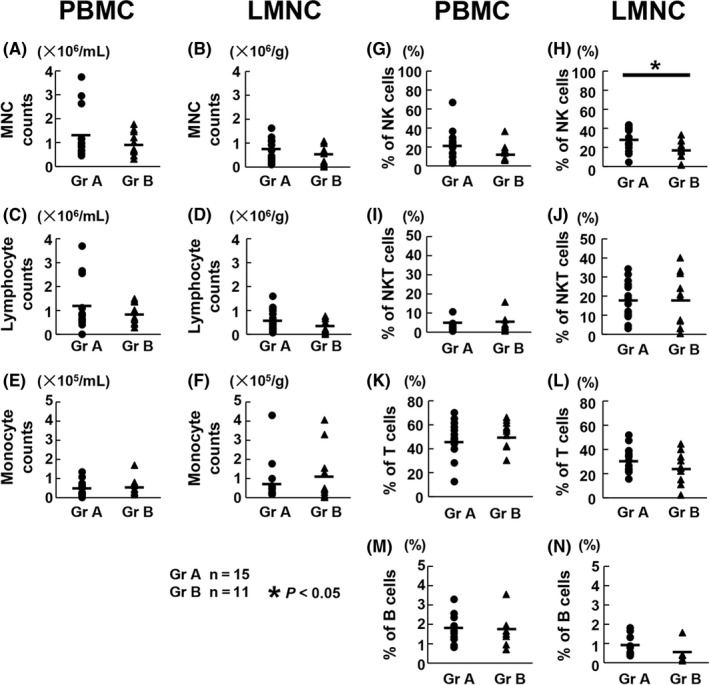
Effects of neoadjuvant chemotherapy on lymphocyte subsets in both peripheral blood mononuclear cells (PBMC) and liver mononuclear cells (LMNC). Numbers and proportions of PBMC and LMNC were analyzed by flow cytometric assay in groups A and B (n = 15 and 11, respectively). A, Comparison of the number of PBMC per mL of blood between the two groups. B, Comparison of the number of LMNC collected by liver perfusion per g of resected liver between the two groups. C‐F, Comparison of the number of lymphocytes and monocytes in PBMC and LMNC between the two groups. G‐N, Comparison of the percentages of natural killer (NK) cells, NKT cells, T cells, and B cells in PBMC and LMNC between the two groups. *P < 0.05

A comparison of the numbers of PBMC, lymphocytes, and monocytes showed no significant differences between groups A and B (Figure 1A,C,E), and no significant differences were observed between the two groups in terms of the numbers of LMNC, lymphocytes, and monocytes collected during liver perfusion from resected livers (Figure 1B,D,F). We compared proportion of NK cells, NKT cells, T cells, and B cells in both PBMC and LMNC between both groups to analyze the effect of neoadjuvant chemotherapy on lymphocyte populations. No significant differences were found in the proportion of NK cells in PBMC (Figure 1G). However, the proportion of NK cells in LMNC was significantly lower in group B than in group A (17.1 ± 10.0% vs 28.2 ± 12.2%; *P* = 0.03; Figure 1H). The proportion of NKT cells, T cells, and B cells in both PBMC and LMNC did not differ significantly between two groups (Figure 1I‐N). Further, we evaluated the correlation of NK and T cells to examine the relationship between innate and adaptive immunities. In both groups, a positive correlation was noted between the number of peripheral blood NK cells and that of T cells. Moreover, although there was a positive correlation between the number of liver NK cells and that of T cells in group B, this was not observed in group A (Figure S1).

### 3.3 Potential augmentation of NK cell activity in the liver of patients receiving neoadjuvant chemotherapy followed by hepatectomy

**Figure 2:**
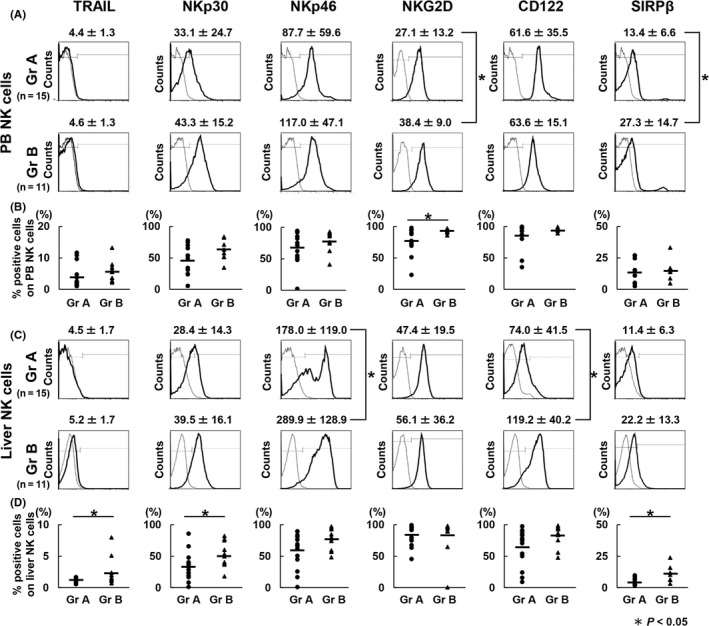
Neoadjuvant chemotherapy increased the expression of activation markers in peripheral blood and liver natural killer (NK) cells. Flow cytometric analysis of peripheral blood mononuclear cells and freshly isolated liver mononuclear cells obtained from liver perfusate after staining with CD3 and CD56 monoclonal antibodies (mAbs) together with additional mAbs was carried out (n = 15 [group (Gr) A]; n = 11 [Gr B]). A, Histograms represent the log fluorescence intensities obtained by staining for tumor necrosis factor‐related apoptosis‐inducing ligand (TRAIL), NKp30, NKP46, natural‐killer group 2, member D (NKG2D), CD122, and signal regulatory protein β (SIRPβ) after gating of CD3^−^ CD56^+^ peripheral blood NK cell subsets obtained from patients. Dotted lines, negative control staining with isotype‐matched mAbs; numbers above each histogram, mean ± SD of the mean fluorescent intensity of targeted molecule expression on peripheral blood NK cells. B, Each point indicates the percentage of peripheral blood NK cells in each group that was positive for TRAIL, NKp30, NKP46, NKG2D, CD122, and SIRPβ expression. C, Histograms representing the log fluorescence intensities obtained by staining for TRAIL, NKp30, NKP46, NKG2D, CD122, and SIRPβ after gating of CD3^−^ CD56^+^ liver NK cell subsets obtained from patients. Dotted lines, negative control staining with isotype‐matched mAbs; numbers above each histogram, mean ± SD of the mean fluorescent intensity of targeted molecule expression on liver NK cells. D, Each point indicates the percentage of liver NK cells in each group that was positive for the TRAIL, NKp30, NKP46, NKG2D, CD122, and SIRPβ expression. *P < 0.05

We assessed phenotypic differences in NK cells, which play a pivotal role in tumor surveillance, to investigate the effect of neoadjuvant chemotherapy on the innate‐immune system in CRLM patients. Mean fluorescent intensity (MFI) of NKG2D and SIRPβ, which is associated with the small transmembrane adapter protein DAP12, transduce stimulatory signals,^26^ and are expressed on activated NK cells^27^ on peripheral blood NK cells, was significantly higher in group B than in group A (*P* = 0.03 and *P* = 0.04, respectively; Figure 2A). Proportion of NKG2D‐positive NK cells was also significantly elevated in PBMC from group B compared with group A (*P* = 0.04; Figure 2B). Proportion of TRAIL‐positive NK cells significantly increased in LMNC from group B compared with that from group A (*P* = 0.03; Figure 2D). Reportedly, TRAIL can induce either apoptosis by Fas‐associated death domain‐dependent mechanism or necrosis through receptor‐interactive peptide‐dependent cascade through the ligation of its death domain‐containing receptors under physiological conditions.^28^ MFI of NKp46 and CD122, known as IL‐2Rβ, on liver NK cells was significantly higher in group B than in group A (*P* = 0.04 and *P* = 0.03, respectively; Figure 2C). Proportion of NKp30 and SIRPβ‐positive NK cells significantly increased in LMNC from group B compared with LMNC from group A (*P* = 0.04 and *P* = 0.02, respectively; Figure 2D). Overall, the expression of cytotoxic and activation molecules in peripheral blood and liver NK cells tended to be enhanced in group B compared with group A. Further, we determined whether the adverse events of neoadjuvant chemotherapy are involved in the functions of NK cells. Notably, in group B, there was no difference in terms of the expression of surface molecules on NK cells between cases with myelosuppression (n = 4) and those without myelosuppression (n = 7) (Figure S2).

**Figure 3:**
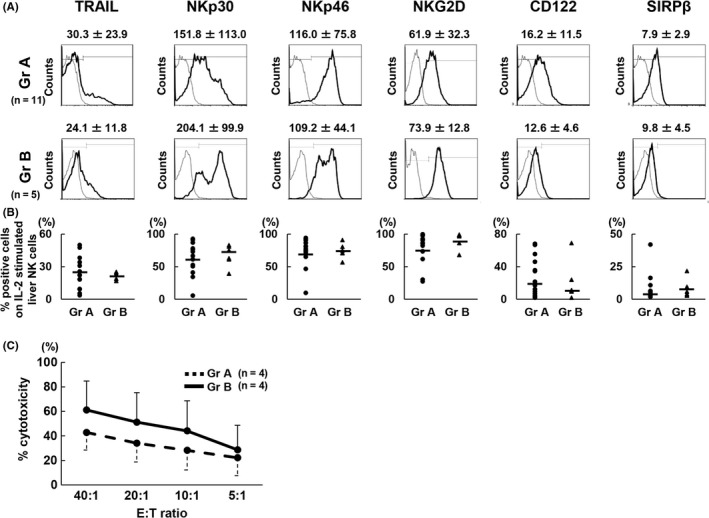
Neoadjuvant chemotherapy did not suppress the cytotoxic activity of cytokine‐stimulated liver mononuclear cells (LMNC). Flow cytometric (FCM) analyses of LMNC cultivated with recombinant interleukin (IL)‐2 for 3 d were carried out after staining with monoclonal antibodies (mAbs) against CD3 and CD56 (n = 11 [Gr A]; n = 5 [Gr B]). A, Histograms represent the log fluorescence intensity obtained by staining for tumor necrosis factor‐related apoptosis‐inducing ligand (TRAIL), NKp30, NKP46, natural‐killer group 2, member D (NKG2D), CD122, and signal regulatory protein β (SIRPβ) after gating of CD3^−^ CD56^+^ IL‐2‐stimulated liver natural killer (NK) cell subsets obtained from patients. Dotted lines, negative control staining with isotype‐matched mAbs; numbers above each histogram, mean ± SD of the mean fluorescent intensity (MFI) of targeted molecule expression on IL‐2‐stimulated liver NK cells. B, Each point indicates the percentage of IL‐2‐stimulated liver NK cells in each group that was positive for TRAIL, NKp30, NKP46, NKG2D, CD122, and SIRPβ expression. C, NK cytotoxic activity of IL‐2‐stimulated LMNC against DLD‐1 target cells was compared between group A (dotted line) and group B (solid line) and analyzed using an FCM‐based cytotoxic assay. All data are expressed as mean ± SD (n = 4 [Gr A]; n = 4 [Gr B])

Then, we investigated the influence of the cytotoxic activity of LMNC against DLD‐1 CRC cells. We stimulated LMNC obtained from each group with IL‐2 and evaluated phenotypic alterations in these cells using the FCM assay. Of note, IL‐2‐stimulated LMNC were used as effector cells rather than freshly isolated LMNC because of the limited level of cytotoxic activity of freshly isolated LMNC (data not shown). IL‐2 stimulation increased the expression of activated molecules such as TRAIL, NKp30, NKp46, and NKG2D on liver NK cells compared to the naive condition in both groups, and the remarkable differences in the expression levels of surface molecules in IL‐2‐stimulated LMNC from both groups were no longer observed (Figure 3A,B). Consequently, the cytotoxic activity of IL‐2‐stimulated LMNC obtained from both groups showed no significant differences (Figure 3C).

### 3.4 Neoadjuvant chemotherapy did not affect the expression levels of NKG2D ligands in liver tumors

**Figure 4:**
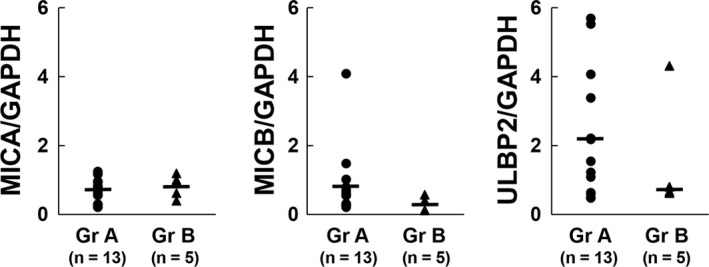
Neoadjuvant chemotherapy did not affect the expression levels of major histocompatibility complex (MHC) class I polypeptide‐related sequence A (MICA), MHC class I polypeptide‐related sequence B (MICB), and UL16‐binding protein 2 (ULBP2) in liver tumors. Expression levels of natural‐killer group 2, member D (NKG2D) ligands (MICA, MICB, and ULBP2) in resected liver specimens of colorectal cancer with liver metastasis (CRLM) from each group were investigated (n = 13 [Gr A]; n = 5 [Gr B]). RNA from liver tumors was extracted and reverse‐transcribed. Relative copy numbers of NKG2D ligands were determined by real‐time PCR using each NKG2D ligand‐specific primer pair and normalized to the expression of GAPDH

We then evaluated the influence of chemotherapy on the expression of tumor‐specific antigens that were theoretically recognized by NK cells on tumors from CRLM patients. Ligands for NKG2D comprised human class I‐like molecules MICA, MICB, and ULBP, which are stress‐induced molecules expressed by tumors of epithelial origin and activate NK cell cytotoxicity through their NKG2D receptor.^29^, ^30^ Although we carried out immunohistochemistry using frozen sections as a preliminary experiment, evaluation of the expression of MICA, MICB, and ULBP2 in tumor tissue was challenging. Hence, we examined the expression levels of these molecules in liver specimens from patients in each group by RT‐PCR. Ligands for NKG2D were evaluable in 13 of 15 samples in group A and in five of 11 samples in group B. However, we could not carry out complete sequencing on other samples because of a lack of DNA or degradation of DNA. Difficulty in extracting DNA from liver tissue because of tumor necrosis accounted for the small number of samples in group B. Furthermore, although we could not exclude the possibility of inconsistencies arising from this limitation, no significant differences were observed in the expression levels of these three targets between the two groups (Figure 4).

### 3.5 No association between NK cell activation marker expression and clinically evaluated chemotherapy response rate

**Figure 5:**
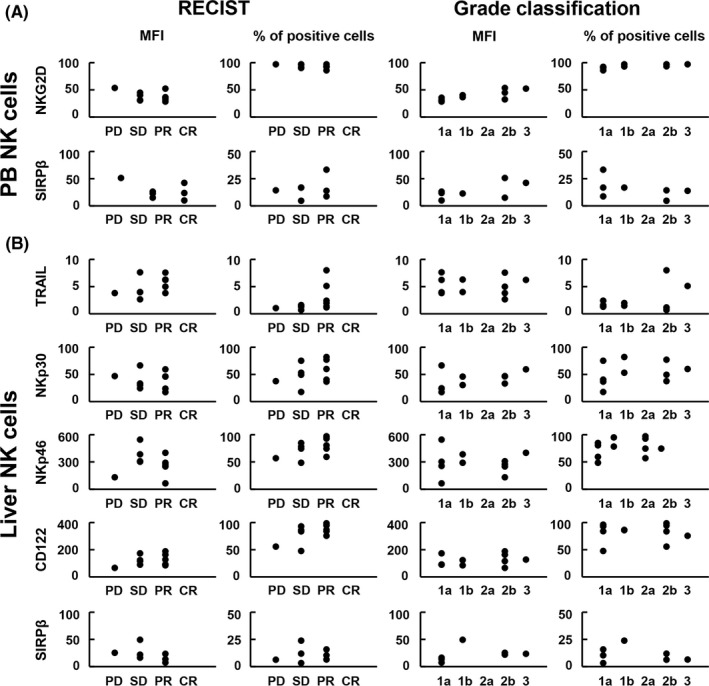
Neoadjuvant chemotherapy did not correlate with the clinical/pathological response rates and the expression levels of surface molecules on natural killer (NK) cells. Clinical evaluation of response by Response Evaluation Criteria In Solid Tumors (RECIST) and histological evaluation of treatment response (grade classification) were carried out in group B (n = 11). A, Relationships between tumor necrosis factor‐related apoptosis‐inducing ligand (TRAIL), natural‐killer group 2, member D (NKG2D), and signal regulatory protein β (SIRPβ) on peripheral blood NK cells and clinical response determined by RECIST or histological treatment response (grade classification) were studied. B, Relationships between TRAIL, NKp30, NKp46, CD122, and SIRPβ on liver NK cells and clinical response determined by RECIST or histological treatment response (grade classification) were studied

To investigate the effect of neoadjuvant chemotherapy on tumor shrinkage, we assessed the correlation between NK cell surface activation markers, such as TRAIL, NKp30, NKp46, NKG2D, CD122, and SIRPβ, in PBMC and LMNC and the chemotherapy response clinically evaluated using RECIST or the histological treatment response (grade classification) were studied in patients receiving neoadjuvant chemotherapy followed by hepatectomy. Evaluation of RECIST in 11 patients showed no CR cases, whereas there were six PR cases (54.6%), four SD cases (36.4%), and one PD case (9.0%). Four cases were classified as grade 1a (36.4%), two cases as grade 1b (18.2%), zero cases as grade 2a (0%), four cases as grade 2b (36.4%) and one case as grade 3 (9.0%). Despite confirming the treatment effect in various cases, we observed no significant correlation between the expression levels of each activated molecule in NK cells and in image‐confirmed or pathological chemotherapy response rates (Figure 5).

## 4 DISCUSSION

Effect of chemotherapeutic agents on immune cell function is considered to be involved in the immunosuppression associated with myelosuppression or drug cytotoxicity.^31^ However, the immune cell function, particularly regarding liver immunity at the local anatomical site with distant CRC metastasis, during chemotherapy, remains unclear. To resolve this clinical question, we separately designed a subanalysis study of a phase II/III randomized clinical trial, which was conducted by the HiSCO group, to investigate whether chemotherapeutic or surgical precedence as a primary treatment has a clinical advantage for treatment of resectable CRLM patients.

To date, several studies have reported correlations between immunosuppressed status and tumor recurrence or patient prognosis in various malignant tumors. Reportedly, preoperative variables, including decreased lymphocyte count, increased monocyte count, and elevated neutrophil‐to‐lymphocyte ratio, are associated with poor prognosis in cancer patients.^32^, ^33^ In addition, perioperative changes in peripheral blood monocyte counts are independent risk factors for OS after hepatectomy and may reflect an immunosuppressive state.^34^ In this study, PBMC and LMNC counts, including those of lymphocytes and monocytes, of the group receiving neoadjuvant chemotherapy were not less than those of the group primarily receiving hepatectomy. Hence, preoperative introduction of bevacizumab combined with CapeOX chemotherapy for the treatment of CRLM may not weaken the immune system function in these patients. Generally, the innate and adaptive immune systems can protect the host against tumor development through immunosurveillance. In this study, we showed a positive correlation between the number of liver NK cells and that of T cells in the group receiving neoadjuvant chemotherapy followed by hepatectomy, possibly indicating the effect of the innate immune system against the adaptive immune system. However, further investigation is warranted to describe the precise role of neoadjuvant chemotherapy in adaptive immunity following NK cell activation in patients with resectable CRLM.

Several investigators have reported that patients with a relatively dense infiltration of malignant tumors by NK cells showed better clinical outcomes.^18^, ^35^, ^36^ Outcomes of NK cell‐target cell interactions are regulated by a fine integrative balance between inhibitory and activating receptors expressed on NK cells and their ligands on target cells.^37^ In addition, several studies have indicated that a majority of CRC show diminished MHC class I expression, making them particularly vulnerable to NK cell‐mediated killing and these patients show survival benefit.^38^, ^39^ However, the precise mechanism of NK cell function in CRLM tissues remains unclear. In the present study, the proportion of peripheral blood NK cells did not significantly differ between the two groups, although the proportion of liver NK cells was significantly lower in the neoadjuvant chemotherapy group. We collected NK cells in the liver by a nondestructive perfusion method to avoid the possibility of enzyme‐induced alteration or disruption of specific epitopes and evaluated the effect of chemotherapy on NK cells in the liver. In this case, the tumor infiltrated mononuclear cells, among which the NK cell fraction might be enriched through potential chemotaxis from the tumor endothelium, are likely to be present in the perfusate, hence the collected LMNC contain tumor‐infiltrated immunocytes. Because >50% of the patients displayed PR in the neoadjuvant chemotherapy group, the number of liver NK cells that infiltrated into the tumor decreased as a result of tumor shrinkage. Consequently, the proportion of NK cells among LMNC possibly reduced in these patients. Another possibility might be that the chemotherapy comprising bevacizumab plus CapeOX directly influences the existence of NK cells in the liver. To address this concern, further investigation may be required.

Efficacy of NK cell‐mediated tumor clearance depends on the type of NK cells that are present in the tissue, or that have migrated to the tumor site from peripheral blood.^40^ Non‐classical MHC class I antigens, such as MICA, MICB, and ULBP, can engage a stimulatory receptor on NK cells comprising a heterodimeric complex of NKG2D/DAP10, a cell‐surface adaptor molecule involved in signal transduction.^29^, ^41^ The activation signal resulting from the engagement of NKG2D‐DAP10 overrides the inhibitory signal from MHC class I molecules leading to target cell lysis.^30^, ^42^ Reportedly, high density of intratumoral NKp46‐expressing NK cells was associated with OS in CRLM patients who received neoadjuvant chemotherapy.^18^ In this study, the expression of activation markers/effector molecules on both peripheral and liver NK cells of patients treated with neoadjuvant chemotherapy was enhanced compared with that in patients who did not receive chemotherapy; however, the proportion of NK cells among LMNC was reduced. Our results indicated increased NKG2D‐expressing peripheral blood NK cells and NKp46‐expressing liver NK cells in the neoadjuvant chemotherapy group. Furthermore, the proportion of TRAIL‐positive NK cells in LMNC significantly increased in the neoadjuvant chemotherapy group. Studies have shown TRAIL to be critical among tumor necrosis factor (TNF) family members in the NK cell‐mediated antitumor function.^20^, ^43^ Moreover, CRC cells are susceptible to TRAIL‐induced apoptosis, both as a single agent of recombinant TRAIL and combined with chemotherapy and targeted therapies.^44^ Thus, giving preoperative chemotherapeutic agents phenotypically activated liver NK cells; however, the underlying mechanism remains unclear. This seems consistent with a previous finding that dying tumor cells treated with chemotherapeutic agents release proinflammatory cytokines that are crucial in the stimulation of protective anti‐cancer immune responses.^45^ Thus, it is possible that neoadjuvant chemotherapy enhances NK cell‐mediated cytotoxicity against tumor cells; however, we could not demonstrate the enhancement of cytotoxic activity in our experimental system. Its clinical significance remains completely unknown. Furthermore, we confirmed the effect of neoadjuvant chemotherapy on NK cells and showed that myelosuppression did not affect at least the expression of surface molecules on NK cells in cases wherein chemotherapy could be continued in the group receiving neoadjuvant chemotherapy followed by hepatectomy. However, the effects of the adverse events of neoadjuvant chemotherapy on intrahepatic immune cells are still unknown.

In addition, NKG2D ligand expression is reportedly correlated with better clinical prognosis in CRC.^46^ In this study, neoadjuvant chemotherapy did not affect the expression of non‐classical MHC class I antigens on CRLM tumor tissues, suggesting that chemotherapeutic agents might not diminish susceptibility to NK cell‐mediated anti‐tumor cytotoxicity. Cytotoxic activity of IL‐2‐stimulated LMNC from the neoadjuvant chemotherapy group was not significantly different from that of the group that received surgery first. These results suggest that chemotherapy does not have a negative impact on intrahepatic immune cells in resectable CRLM patients.

Recently, a complete pathological response was shown to be correlated with high rates of the OS and PFS in advanced CRC patients who had undergone neoadjuvant and conversion chemotherapy before resection of CRLM.^47^ In the present study focusing on the early limited phase until hepatectomy, we found no significant correlation between the expression strength of activating markers on NK cells and the image‐confirmed and pathological chemotherapy response rates at the time of surgical treatment. The main HiSCO‐01 trial is still under long‐term follow‐up observations, thereby not allowing us to evaluate the correlation between the expression strength of the activating markers on NK cells and survival benefits.

In summary, the present study suggests that neoadjuvant chemotherapy for the treatment of resectable CRLM induces the activation of peripheral blood and liver NK cells; however, its clinical significance remains unknown.

## DISCLOSURE

Funding: This work was partly supported by a Grant‐in‐Aid for Research on Hepatitis from the Japan Agency for Medical Research and Development (AMED: 16fk0210107 h0001).

Conflicts of Interest: The protocol for this research project was approved by a suitably constituted Ethics Committee of the institution and it conforms to the provisions of the Declaration of Helsinki. Committee of Hiroshima University Clinical Research Ethics Review, Approval No. Clinical 186. Written informed consent was obtained from subjects and/or guardians.
