# Supplementary figures and images for: Effect of bevacizumab plus XELOX (CapeOX) chemotherapy on liver natural killer cell activity in colorectal cancer with resectable liver metastasis
Source: Ann Gastroenterol Surg. 2018 Jul 18;2(5):383–93. doi: 10.1002/ags3.12195 (PMC6139723; doi:10.1002/ags3.12195)

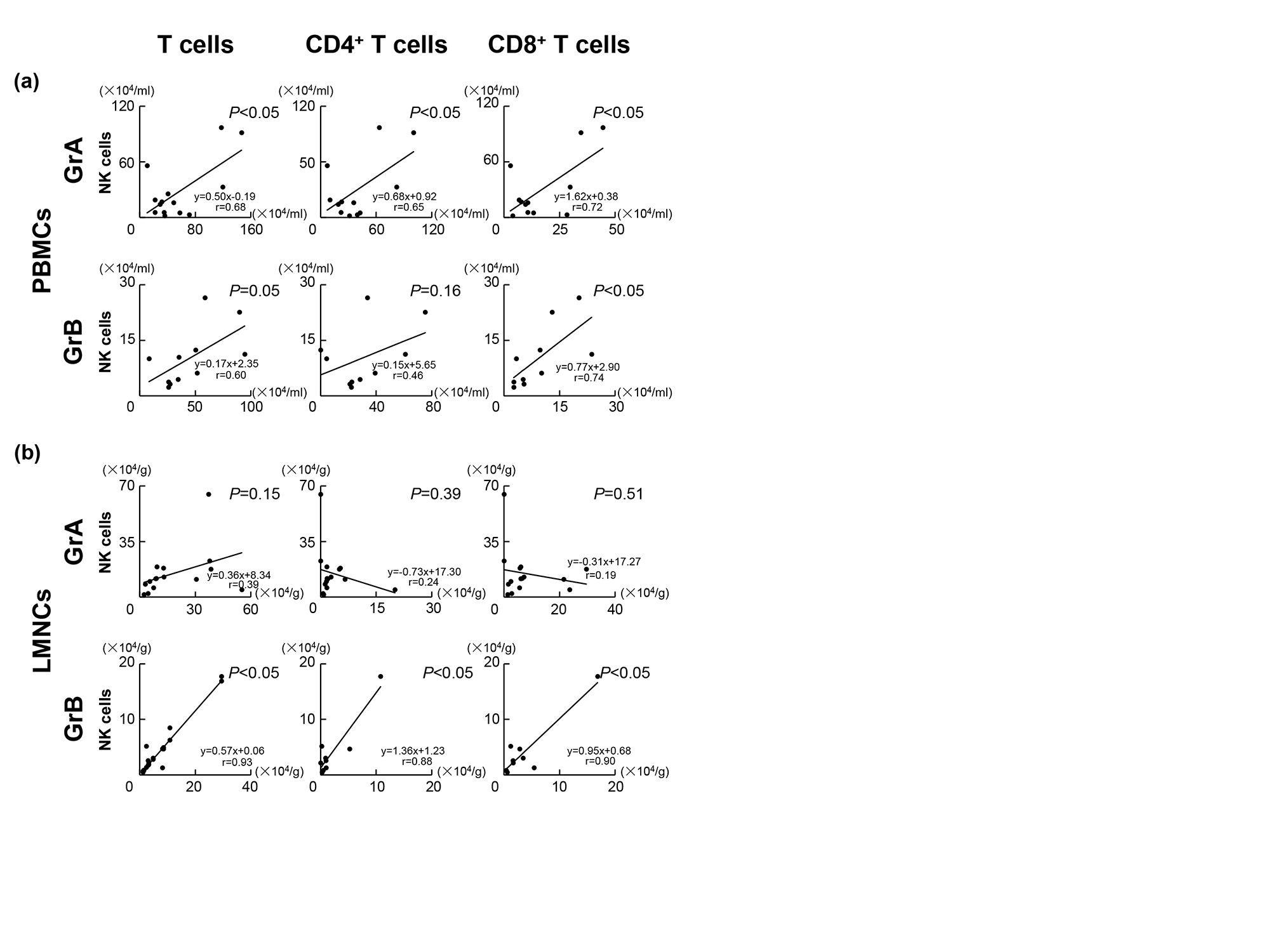

Supplement: Supplementary file 1 [file AGS3-2-383-s001.tif]

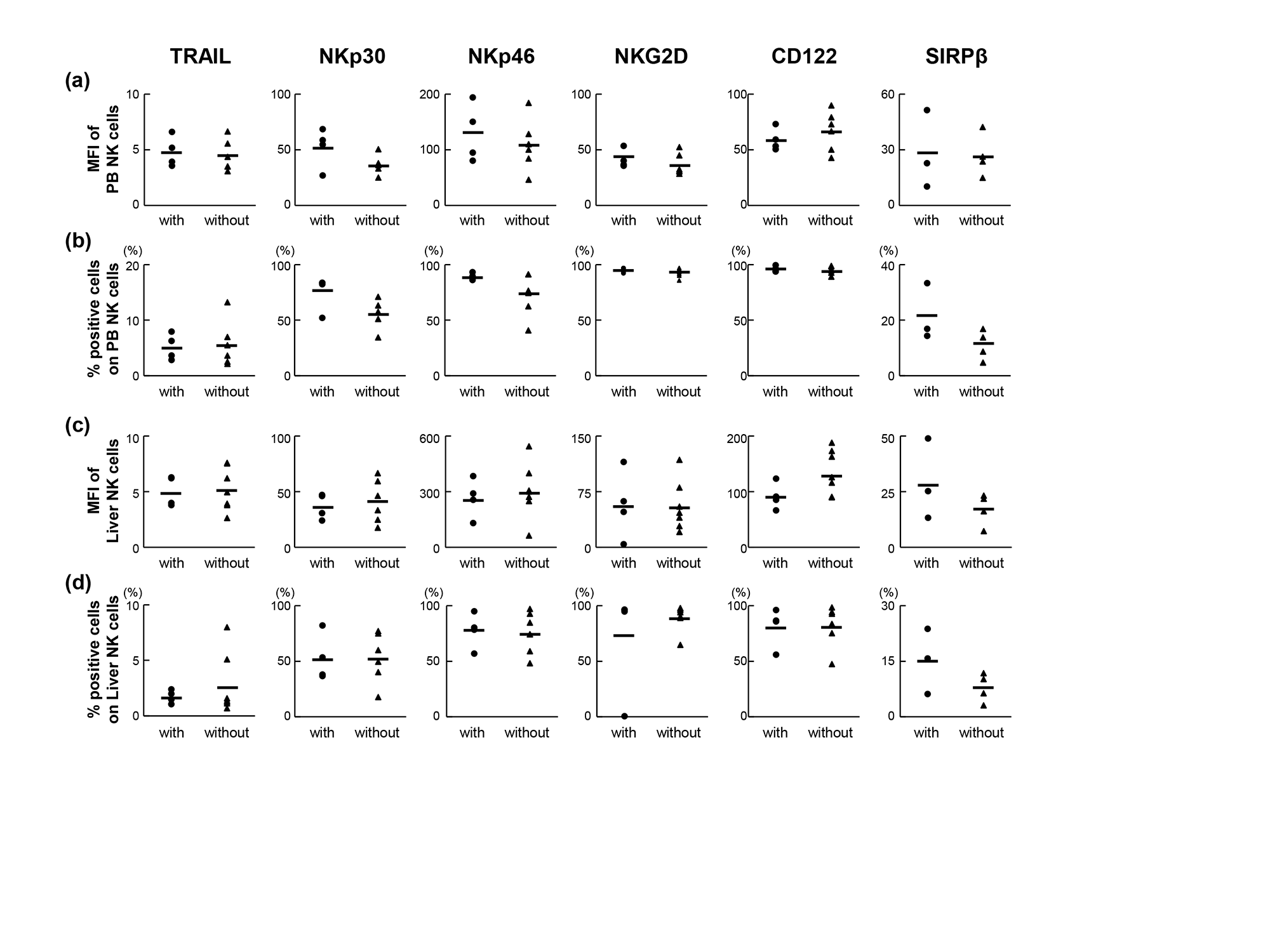

Supplement: Supplementary file 2 [file AGS3-2-383-s002.tif]
